# Interplay between Tumor Mutational Burden and Mutational Profile and Its Effect on Overall Survival: A Pilot Study of Metastatic Patients Treated with Immune Checkpoint Inhibitors

**DOI:** 10.3390/cancers14215433

**Published:** 2022-11-04

**Authors:** Camila B. Xavier, Carlos Diego H. Lopes, Beatriz M. Awni, Eduardo F. Campos, João Pedro B. Alves, Anamaria A. Camargo, Gabriela D. A. Guardia, Pedro A. F. Galante, Denis L. Jardim

**Affiliations:** 1 Oncology Center, Hospital Sírio-Libanês, São Paulo 01308-050, Brazil; 2 Falconi Consultants for Results, São Paulo 045043-011, Brazil; 3 Molecular Oncology Center, Instituto de Ensino e Pesquisa, Hospital Sírio-Libanês, São Paulo 01308-050, Brazil

**Keywords:** tumor mutational burden, immune checkpoint inhibitors, immunotherapy, mutation profile, NGS

## Abstract

**Simple Summary:**

Recently, patients with high-TMB tumors received agnostic FDA approval to be treated with pembrolizumab. However, some high-TMB patients do not show clinical benefits from this strategy. In this manuscript, we investigated a large cohort of 488 patients with TMB ≥ 10 mut/Mb treated with the following immune checkpoint inhibitors (ICIs), and correlated the clinical outcomes with the distinct somatic mutational profile of tumors: monoclonal antibody directed against programmed cell death protein-1 or monoclonal antibody directed against programmed cell death ligand 1 (anti-PD-1/anti-PD-L1); monoclonal antibody directed against cytotoxic T lymphocyte-associated antigen (anti-CTLA-4); combined treatment regimen including one anti-PD-1/anti-PD-L1 and one anti-CTLA-4 (ICIs combination). We know that some genomic alterations in TMB-high patients are already documented to help define prognosis and outcomes during immunotherapy. Conversely, other variables, such as MSI status, age, or gender, were not important to predict response to ICI treatment in this scenario, which could hypothesize the presence of a response prediction hierarchy. Thus, we believe that our manuscript is of broad interest to the general oncology community and can be used to better select patients for ICI treatment.

**Abstract:**

Purpose: Solid tumors harboring tumor mutational burden (TMB) ≥10 mutations per megabase (mut/Mb) received agnostic approval for pembrolizumab. This work aims to analyze the somatic mutational profile’s influence on the outcomes of patients with TMB-high tumors treated with immune checkpoint inhibitors (ICIs). Methods: This post-hoc analysis evaluated clinical and molecular features of patients with solid tumors treated with ICIs that could be either monoclonal antibody directed against programmed cell death protein-1 or monoclonal antibody directed against programmed cell death ligand 1 (anti-PD-1/anti-PD-L1), monoclonal antibody directed against cytotoxic T lymphocyte-associated antigen (anti-CTLA-4) or a combined treatment regimen including one anti-PD-1/anti-PD-L1 and one anti-CTLA-4 (ICIs combination). We performed OS analysis for TMB thresholds of ≥10, ≥20, and <10 mut/Mb. We assessed OS according to the mutational profile for a TMB ≥ 10 mut/Mb cutoff. For genes correlated with OS at the univariate assessment, we conducted a Cox multivariate analysis adjusted by median TMB, sex, age, microsatellite instability (MSI), and histology. Results: A total of 1661 patients were investigated; 488 with a TMB ≥10 mut/Mb (29.4%). The median OS was 42 months for TMB ≥10 or 20 mut/Mb, and 15 months for TMB <10 mut/Mb (*p* < 0.005). Among TMB ≥10 mut/Mb patients, mutations in E2F3 or STK11 correlated with worse OS, and mutations in NTRK3, PTPRD, RNF43, TENT5C, TET1, or ZFHX3 with better OS. These associations were confirmed with univariate and multivariate analyses (*p* < 0.05). Melanoma histology and TMB above the median endowed patients with better OS (*p* < 0.05), while MSI status, age, and gender did not have a statistically significant effect on OS. Conclusion: Combining TMB and mutation profiles in key cancer genes can better qualify patients for ICI treatment and predict their OS.

## 1. Introduction

Tumor mutational burden (TMB) was correlated with response to immune checkpoint inhibitors (ICIs) in a retrospective cohort, including 1661 patients treated with ICIs, that could be either monoclonal antibody directed against programmed cell death protein-1 or monoclonal antibody directed against programmed cell death ligand 1 (anti-PD-1/anti-PD-L1), monoclonal antibody directed against cytotoxic T lymphocyte-associated antigen (anti-CTLA-4) or a combined treatment regimen including one anti-PD-1/anti-PD-L1 and one anti-CTLA-4 (ICIs combination). Among all patients, higher somatic TMB, defined as the highest 20% in histology, was a predictor of better overall survival (OS) [1]. Subsequently, a prospective analysis from the phase II KEYNOTE-158 trial stated that a TMB of at least 10 somatic tumor mutations per megabase (mut/Mb) was associated with a higher proportion of objective response rates (ORR) to pembrolizumab monotherapy [2]. These results led to the FDA agnostic approval of pembrolizumab for TMB-high (≥ 10 mut/Mb) patients. However, 42% of patients presenting with high TMB do not respond to ICIs, indicating the need for better patient selection in this setting. Currently, a variety of clinical and molecular factors may have important roles in modulating the tumor response to ICIs [3]. Factors such as MHC diversity [4,5] and concomitant molecular alterations influence TMB as a biomarker for immunotherapy. To better qualify patients with TMB ≥ 10 mut/Mb for ICI treatment, we investigated their mutation profiles and correlated molecular alterations with their survival outcomes when treated with ICIs.

## 2. Materials and Methods

### 2.1. Study Design

We collected genomic and survival data from 1661 patients [1] treated with at least one dose of ICIs (anti-PD-1/anti-PD-L1, anti-CTLA-4 or ICIs combination and retrieved their mutation profiles (MSK-IMPACT). All the data collected was available on the public database https://www.cbioportal.org/ (accessed on 1 July 2021) [6,7]. MSK-IMPACT assay identified somatic exonic mutations in a predefined subset of 468 cancer-related genes (earlier versions included 341 or 410 genes), by using both tumor-derived and matched germline normal DNA. TMB was determined by the number of nonsynonymous somatic mutations. Firstly, a validation analysis correlated predefined percentiles used in the original publication [1] with the absolute thresholds of ≥10, ≥20, and <10 mut/Mb. The TMB cutoff of ≥10 mut/Mb included the ≥20 mut/Mb samples. A separate analysis for the 10–20 mut/Mb cutoff was not performed. We pursued this higher cutoff for patient selection refinement. Further, for a TMB ≥10 mut/Mb cutoff, we selected mutations that occurred in at least 5 patients and assessed OS according to somatic mutational profiles in key cancer genes. For gene mutations exhibiting a positive correlation with OS (*p* < 0.05) at the univariate assessment, we conducted a Cox multivariate analysis adjusted by median TMB, sex, median age, microsatellite instability (MSI) status, and histology. Since MSI status was not available in the current database, an individual assessment of somatic mutations in *MLH1, MSH2, MSH6, PMS2*, and *SETD2* was used as a surrogate for MSI estimation [8]. A flowchart encompassing the study design is available in Supplementary Materials Figure S1.

### 2.2. Statistical Analysis

Overall survival (OS) for all patients who received at least one dose of ICIs was estimated using the Kaplan-Meier method. The Cox regression model was used to define the hazard ratios (HRs) for death, and a log-rank test was used to compare the results (95% confidence intervals for all analyses). The Python Lifelines package (version 0.26.4) [9] was used for Kaplan–Meier and Cox analyses.

For a TMB ≥10 mut/Mb cutoff (N = 488), we assessed OS regarding the mutational status of each gene mutation found in at least 5 patients (N = 392). For all genes exhibiting a correlation with survival considering a standard alpha-error level (*p* < 0.05), a Cox multivariate analysis was also conducted using Reboot [10]. The adjustment variables included sex, median age, microsatellite instability (MSI) status, TMB under or above the median TMB of the cohort (20 mut/Mb), and tumor histology (non-small cell lung cancer (NSCLC), melanoma, bladder cancer, and colorectal cancer).

## 3. Results

### 3.1. Patients’ Characteristics

A total of 1661 patients (11 cancer types) were included. The median follow-up was 19 months (range 0–80). The top three more incident tumors were NSCLC (21.1%), melanoma (19.3%), and bladder cancer (12.9%). Of all samples, 488 (29.4%) harbored a TMB of ≥ 10 mut/Mb. When the original criteria of top 20% in each histology was adopted, only 332 (20%) patients were considered to be TMB-high. Of these patients, 279 (84%) presented TMB ≥ 10 mut/Mb. MSI surrogates were detected in 42 (29.1%) of the cases. The ICI class delivered was anti-PD-1/anti-PD-L1 in 78.7%, anti-CTLA-4 in 6%, and ICI combination in 15.4% of the cases. Genes most frequently harboring genomic alterations (incidence >10% of all tumor samples) were *TP53*, *TERT*, *KMT2D*, *KRAS*, *PIK3CA*, *ARID1A*, *NF1*, and *PTPRT*. Sample characteristics are summarized in Table 1.

**Figure 1:**
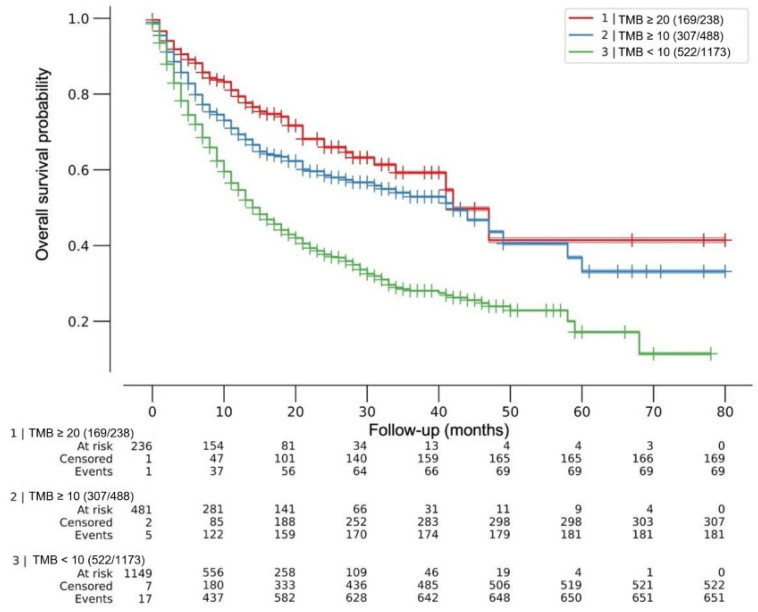
Effect of tumor mutational burden (TMB) on overall survival after ICI treatment. Kaplan–Meier (KM) curves for patients with tumors within each TMB predefined cutoff. Overall survival was from the first dose of ICIs. Median OS was 42 months for both TMB ≥ 20mut/Mb and TMB ≥ 10 mut/Mb, and 15 months for TMB < 10 mut/Mb, multivariate log-rank *p* < 0.005. Cox regression HRs for death were 0.44 (95% CI 0.34–0.56) and 0.57 (95% CI 0.49–0.67) for TMB ≥ 20 mut/Mb and ≥ 10 mut/Mb, respectively (*p* < 0.005). No difference was observed in death risk between cohorts with TMB 10 mut/Mb or less and 1 mut/Mb or less (HR 0.96; 95% CI 0.74–1.24; *p* = 0.73). ICIs = immune checkpoint inhibitors.

After a maximum follow-up of 80 months, the median OS was 42 months for both TMB ≥10 mut/Mb and TMB ≥20 mut/Mb, and 15 months for TMB <10 mut/Mb, multivariate log-rank *p* < 0.005. The HRs for death were HR 0.44 (95% CI 0.34–0.56) and 0.57 (95% CI 0.49–0.67) for TMB ≥20 mut/Mb and ≥10 mut/Mb, respectively (*p* < 0.005). No difference was observed in death risk between cohorts with TMB 10 mut/Mb or less and 1 mut/Mb or less (HR 0.96; 95% CI 0.74–1.24; *p* = 0.73). Figure 1.

### 3.2. Single Gene Alterations and Implications for Survival 

A total of 392 genes, having somatic mutations that were found in at least five patients were eligible for this analysis. Twenty-seven genes exhibited a statistically significant correlation with OS after ICI treatment when only tumors with TMB ≥ 10 mut/Mb were analyzed. Among them, five genes showed reduced (*p* < 0.05) OS on ICI treatment (*STK11*, *KEAP1*, *CIC, E2F3*, and *TP53*), whereas 22 genes were associated with better (*p* < 0.05) OS (*NTRK3, TERT*, *NOTCH3*, *RNF43*, *TET1*, *PTPRD*, *NCOA3*, *TENT5C*, *ZFHX3*, *RIT1*, *CCNE1*, *PPM1D*, *GATA2*, *ALK*, *DNMT1*, *PTPRT*, *MET*, *EPHA7*, *BCL6*, *SMO*, *CDK6* and *MED12)*. The individual OS for each gene is available in Supplementary Table S1. 

### 3.3. Multivariate Analysis of Individual Gene Alterations in High TMB Patients

**Figure 2:**
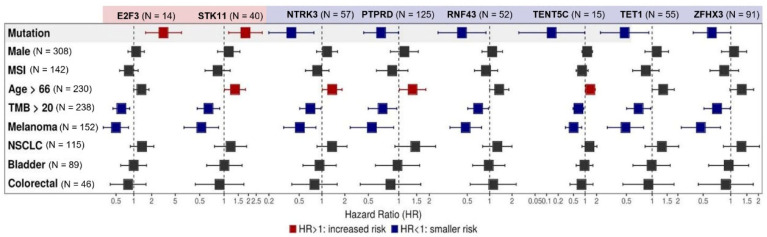
Multivariate analysis of individual gene alterations in high TMB patients. Forest plot for overall survival (OS) in multivariate analysis. Adjustment variables included TMB under or above the median of 20 mut/Mb, sex, median age, microsatellite instability (MSI) status, and tumor types (non-small cell lung cancer (NSCLC), melanoma, bladder cancer, and colorectal cancer). Mutations in STK11 (N = 40, HR 1.84 (95% CI, 1.14 - 2.97) and E2F3 (N = 14, HR 3.17 (95% CI, 1.58–6.38]) were related to worse survival (*p* < 0.05), while mutations in NTRK (N = 57, HR 0.39 95% CI, 0.20–0.78), PTPRD (N = 125, HR 0.67 95% CI, 0.45–0.99), RNF43 (N = 52, HR 0.42 95% CI, 0.2015–0.89), TENT5C (N = 15, HR 0.14 95% CI, 0.02–0.98), TET1 (N = 55, HR 0.48 95% CI, 0.25–0.91), and ZFHX3 (N= 91, HR 0.62 95% CI, 0.39–0.99) were associated with better OS. Melanoma histology and TMB above the median TMB of the cohort (20 mut/Mb) endowed patients with better OS (*p* < 0.05). MSI status, age, and gender did not have a consistent statistically significant effect on OS.

Among the 27 genes exhibiting a correlation with OS (*p* < 0.05) at the univariate assessment, multivariate analysis confirmed a correlation between mutations in *STK11* (N = 40, HR 1.84 (95% CI, 1.14–2.97) and *E2F3* (N = 14, HR 3.17 (95% CI, 1.58–6.38]) and worse survival (*p* < 0.05). Mutations in *NTRK3* (N = 57, HR 0.39 95% CI, 0.20–0.78), *PTPRD* (N = 125, HR 0.67 95% CI, 0.45–0.99), *RNF43* (N = 52, HR 0.42 95% CI, 0.2015–0.89), *TENT5C* (N = 15, HR 0.14 95% CI, 0.02–0.98), *TET1* (N = 55, HR 0.48 95% CI, 0.25–0.91), and *ZFHX3* (N= 91, HR 0.62 95% CI, 0.39–0.99) were associated with better OS. When evaluated concurrently with the mutational profile, histology did not play a relevant role in the response to ICIs, except for melanoma, which endowed patients with better OS (*p* < 0.05). TMB above the median TMB of the cohort (20 mut/Mb) was also related to a better OS for all gene mutations (*p* < 0.05). In addition, MSI status, age, and gender did not have a consistent statistically significant effect on OS, as seen in Figure 2. KM curves for each gene related to survival after Cox multivariate analysis can be found in Supplementary Materials Figure S2.

## 4. Discussion

Despite prior data demonstrating that higher TMB correlates with responses to ICIs, the use of TMB as a predictive biomarker for OS still has limitations [3]. In the original publication [1], both the TMB evaluation as a continuous variable and the binary cutoff of the top 20% TMB within each histology, with adjustment for cancer type, age, drug class of ICI, and year ICI treatment started reduced the chance of death across multiple cancer types (HR of 0.99 and 0.61, respectively, *p* < 0.01). This data accorded with our findings, which validated and reinforced the predictive value of TMB ≥ 10 mut/Mb (HR of 0.57, *p* < 0.005) for response to ICI treatment. A stricter TMB cutoff of ≥ 20 mut/Mb could refine patient selection (HR 0.44, *p* < 0.005). Conversely, according to Marabelle et al. [2], only a not-statistically significant difference in estimated 3-year OS was noted between the TMB-high/low groups (32% vs. 22%), and more than half of the patients died regardless of TMB status at the 3-month landmark. In both cases, the use of additional clinical and molecular features could have provided better patient selection for ICI treatment.

While the Keynote-158 pivotal study, that led to Pembrolizumab regulatory approval, included small numbers of less common and often immune-refractory tumors (anal, biliary, cervical, endometrial, mesothelioma, neuroendocrine, salivary, thyroid, vulvar and small-cell lung cancers), the study cohort evaluated in our study reflected the real world epidemiology of ICI use for more frequent tumors, such as in NSCLC, melanoma, bladder cancer, and colorectal cancers.

Regarding genes with somatic mutations, some of them were still less frequently mutated, and the statistical positive association could not be translated to the clinical scenario. Examining examples separately, *CCND3* mutation occurred in 2 cases: one patient with melanoma (concurrent *KIT* and *BRAF* non-V600 mutations) and a second with NSCLC (concurrent *TP53 mutation*), and within two months of follow-up both of these patients had died. Mutation in *TIMM8B* occurred in only one subject with colorectal cancer (TMB of 52.14 mut/Mb and concurrent *BRAF* V600E mutation), and the patient died within one month of PD-1 inhibitor monotherapy. Mutations in *CDKN2B* occurred in one case of cancer of unknown primary site (with concurrent *TP53* mutation), one patient with colorectal cancer, and a third case with melanoma. Within 8 months of follow-up, all the patients were dead. Although the selection of mutations occurring in n > 5 patients for the univariate analysis was arbitrary, we aimed to increase data liability. The complexity of antitumor immune responses is reflected in the absence of a universal biomarker to predict survival benefit from ICIs [11] and an integrative analysis of clinical and molecular variables might better guide patient selection for ICI treatment [12,13].

Concerning MSI status, Goodman et al. [14] evaluated 148,803 tumor samples for TMB and MSI status. Overall, 18.3% of TMB-high tumors harbored MSI, which was less than the finding of 29.1% in our cohort. Although, in both cases, microsatellite stable MSS-/TMB-high amounts for a subgroup of cancers were considerably larger than the MSI subset. Exploratory univariate analyses performed in our TMB-high cohort identified that patients with TMB-high/MSI tumors exhibited better OS outcomes when compared with TMB-high/MSS tumors (median OS 42 vs 19 months; *p* < 0,05). Cox HR for death was 0.77 (95% CI 0.60–0.98; *p* = 0.04), as seen in Supplementary Materials Figures S3 and S4. Further, multivariate analysis to detect MSI influence on survival in TMB-high tumors was negative for all genes listed, which could hypothesize the presence of a response prediction hierarchy being greater for specific gene mutations than MSI. These findings were supported by the recently presented results of the CheckMate-848 study [15], a phase 2 trial that tested Nivolumab with or without Ipilimumab for advanced or metastatic TMB-high solid tumor treatment. Exploratory analysis of the trial regarding the ORR among the TMB-high cohort showed that the responses were observed regardless of the MSI status. MSI was detected in 25 (2.5%) MSI-evaluable tissue samples. ORR for these patients ranged from 33.3% to 55.6% with Nivolumab and Nivolumab plus Ipilimumab, respectively. For MSS tumors, responses ranged from 26.9% to 29.1% with the same ICI regimens. Our multivariate analysis did not find any clinical feature, except for melanoma histology (19.3% of samples studied), that could interfere with survival outcomes after ICI treatment. This melanoma enrichment could also justify a lack of correlation of survival and MSI status, as previous data showed the opposite [16].

Notably, we identified two mutated genes related to poor survival: *STK11* and *E2F3*. Interestingly, there was stronger evidence supporting ICIs resistance related to the *STK11* gene alterations [17]. The STK11 mutation-induced downregulation of immune checkpoint regulating proteins, like PD-L1 and T-cell chemokines, favored a ‘‘cold’’ immunosuppressive tumor microenvironment (TME) and contributed to the exclusion of inflamed immune cells, such as CD4+ T cells, CD8+ T cells, natural killer (NK) cells, and Macrophage type 1 (M1), from driving the tumor immune escape [18]. In accordance, the cell-cycle promoter *E2F3* is a well described tumoral poor prognosis factor and associated with a low immune signature score when amplified [19] (p. 3).

Exploring gene mutations related to better OS, NTRK3 protein expression was previously positively associated with higher tumor immune and stromal scores, a great variety of immune lymphocytes, improved immune response, and, ultimately, with better survival. Accordingly, *NTRK3* might be a novel biomarker for ICI outcomes in selected tumor types [20] (p. 3). The *PTPRD* gene encodes protein tyrosine phosphatase receptor type D (PTPRD), which is related to an increased mRNA expression of JAK1 and STAT1 subsequently attracting T cells through chemokines overexpression. Amidst NSCLC, *PTPRD* mutations endowed patients that received ICIs with better OS [21]. The effect of the mutation in the *RNF43* gene in the TME was evaluated by Zhang et al. [22] (p. 43). Their computed analysis of tumor infiltrating immune cells identified an increment in CD8+ T cells, M1, NK, and total T cells compared with the wild type *RNF43* group. The *TENT5C gene* (which is interchangeable with *FAM46C*) was recently found to be a possible predictor of ICI efficacy, as FAM46C expression was correlated with the abundance of CD4+ T cells, CD8+ T cells, and plasma B lymphocytes in the TME. FAM46C expression was positively correlated with immune chemokines and immune chemokine receptors in most tumors [23]. For the *TET1* gene, previous studies found that tumor infiltrating lymphocytes, particularly the cytotoxic ones, were more abundant among the TET1-mutated tumors. The neoantigen load was higher in this group, indicating that TET1 mutations were correlated with enhanced tumor immunogenicity [24] (p. 1). Finally, for the *ZFHX3* gene, Zhang et al. hypothesized that *ZFHX3*-mutated tumors harbored increased expression of antigen-presentation-related molecules, stimulating immune-related ligands, receptors and chemokines. Furthermore, mRNA analysis of these tumors revealed significantly increased immune checkpoint gene profiles [25]. All the mechanisms described above are potential explanations for our findings, and prospective literature data comprising this molecular selection for ICI use is still lacking.

Our study has limitations, including its retrospective nature based on clinical and molecular data available in a public online database, making it a pilot study. The cohort was also biased toward heterogeneous tumor phenotypes and hypermutated tumors (NSCLC, melanoma, and bladder cancer) [13], which could explain the higher proportion of samples with TMB ≥10 mut/Mb (N 488, 29.4%) and would impact OS results. Second, some clinical information was not available, including objective responses to ICIs, preventing us from establishing associations. Although all patients selected received at least one dose of ICI, we could not access data regarding ICI dosing or frequency. Data regarding previous and posterior treatments or lifestyle were also not available. All these aspects might have influenced the OS results, and the potentially dynamic assessment of TMB over the disease course. Third, we did not include a cohort of TMB-high patients not receiving ICIs; hence, a pure prognostic role of the genes described here cannot be ruled out and future studies can explore their predictive value. In addition, we used mutation in DNA repair genes to define MSI [6], not being able to state that all these patients had microsatellite instability phenotypes.

## 5. Conclusions

Taken together, with this pan-cancer analysis, our findings demonstrate that not only high TMB, but also its combination with somatic mutational profile in some specific genes, can be a predictor of survival benefit from ICI treatment. Although prospective trials are still needed, combining information of TMB and mutation profiles in key cancer genes can be decisive to better qualify patients for ICI treatment.

## Figures and Tables

**Table 1:** Sample characteristics.

	TMB < 10 mut/Mb	TMB ≥ 10 mut/Mb	Total
**N (%)**	1173 (70.6)	488 (29.4)	1661 (100)
**Tumor Type N (%)**			
NSCLC	235 (20)	115 (23.6)	350 (21.1)
Melanoma	168 (14.3)	152 (31.1)	320 (19.3)
Bladder	126 (10.7)	89 (18.2)	215 (12.9)
Renal cell carcinoma	149 (12.7)	2 (0.4)	151 (9.1)
Head and neck cancer	111 (9.5)	28 (5.7)	139 (8.4)
Esophagogastric cancer	107 (9.1)	19 (3.9)	126 (7.6)
Glioma	108 (9.2)	9 (1.8)	117 (7)
Colorectal cancer	64 (5.5)	46 (9.4)	110 (6.6)
Unknown primary cancer	64 (5.5)	24 (4.9)	88 (5.3)
Breast cancer	41 (3.5)	3 (0.6)	44 (2.6)
Skin (non-melanoma)	0 (0)	1 (0.2)	1 (0.1)
**Median age (years)**	62	66	
**Sex**			
Female	447 (38.1)	180 (36.9)	627 (37.8)
Male	726 (61.9)	308 (63.1)	1034 (62.2)
**MSI (%)**	72 (6.1)	142 (29.1)	214 (12.9)
**ICIs type**			
anti-PD-1/anti-PD-L1	933 (79.5)	374 (76.6)	1307 (78.7)
ANTI-CTLA-4	57 (4.9)	42 (8.6)	99 (6)
ICIs combination	183 (15.6)	72 (14.8)	255 (15.4)
**Most frequently found mutations (incidence >10% of all samples) N (%)**			
*TP53*	467 (39.8)	271 (55.5)	738 (44.4)
*TERT*	256 (21.8)	263 (53.9)	519 (31.2)
*KMT2D*	67 (5.7)	169 (34.6)	236 (14.2)
*KRAS*	138 (11.8)	88 (18)	226 (13.6)
*PIK3CA*	104 (8.9)	96 (19.7)	200 (12)
*ARID1A*	59 (5.0)	131 (26.8)	190 (11.4)
*NF1*	54 (4.6)	129 (26.4)	183 (11)
*PTPRT*	48 (4.1)	126 (25.8)	174 (10.5)

N = number; NSCLC = non-small cell lung cancer; MSI = microsatellite instability; ICIs = immune checkpoint inhibitors; anti-PD-1/anti-PD-L1 = monoclonal antibody directed against programmed cell death protein-1 or monoclonal antibody directed against programmed cell death ligand 1; ANTI-CTLA-4 = monoclonal antibody directed against cytotoxic T lymphocyte-associated antigen; ICIs COMBINATION = combined treatment regimen including one anti-PD-1/anti-PD-L1 and one ANTI-CTLA-4.

## Data Availability

All the data collected was available on the public database https://www.cbioportal.org/ (accessed on 1 July 2021).

## Supplementary Materials

The following supporting information can be downloaded at: https://www.mdpi.com/article/10.3390/cancers14215433/s1, Figure S1: Study design flowchart; Table S1: Individual survival data for genes at univariate analysis; Figure S2: Kaplan–Meier (KM) curves for each gene related with survival after Cox multivariate analysis; Figure S3 and Figure S4: Effect of Microsatellite Instability (MSI) on overall survival after ICI treatment.
