# Supplementary material for: Interplay between Tumor Mutational Burden and Mutational Profile and Its Effect on Overall Survival: A Pilot Study of Metastatic Patients Treated with Immune Checkpoint Inhibitors
Source: Cancers (Basel). 2022 Nov 4;14(21):5433. doi: 10.3390/cancers14215433 (PMC9657500; doi:10.3390/cancers14215433)
Supplement: Supplementary file 1 [file cancers-14-05433-s001.zip › cancers-1911573-supplementary.pdf]

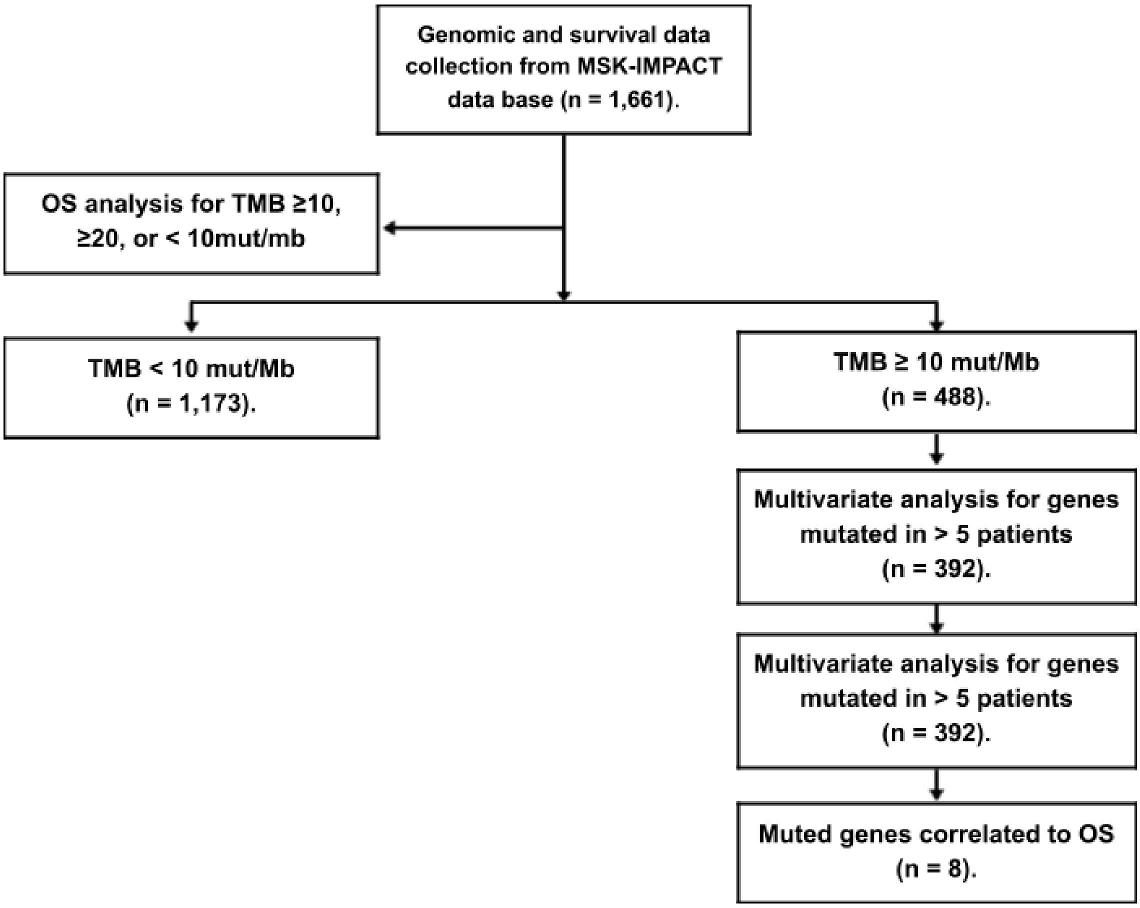

Figure S1. Study design flowchart. OS = overall survival; TMB = tumor mutational burden; mut/Mb = mutations per megabase; MSI = microsatellite instability.

Table S1. Individual survival data for genes at univariate analysis.

Gene	Patients (N)	Median OS (months)	P value
		STK11	
mut	40	7	0.00004
wt	448	44	
		KEAP1	
mut	52	13	0.002
wt	436	44	
		CIC	
mut	49	41	0.02
wt	439	42	
		E2F3	
mut	14	12	0.03
wt	474	44	
		TP53	
mut	271	28	0.03
wt	217	47	
		NTRK3	
mut	57	NR	0.0009
wt	431	36	
		TERT	
mut	263	49	0.002
wt	225	34	
		NOTCH3	
mut	77	47	0.003
wt	411	41	
		RNF43	
mut	52	NR	0.004
wt	436	36	

	TET1		
mut	55	NR	0.005
wt	433	36	
	PTPRD		
mut	125	NR	0.006
wt	363	41	
	NCOA3		
mut	28	NR	0.008
wt	460	41	
	TENT5C		
mut	15	NR	0.008
wt	473	41	
	ZFHX3		
mut	91	NR	0.01
wt	397	41	
	RIT1		
mut	11	NR	0.02
wt	477	42	
	CCNE1		
mut	9	NR	0.03
wt	479	42	
	PPM1D		
mut	21	NR	0.03
wt	467	42	
	GATA2		
mut	12	NR	0.03
wt	476	42	
	ALK		
mut	73	42	0.03

wt	415	36	
	DNMT1		
mut	39	NR	0.04
wt	449	41	
	PTPRT		
mut	126	44	0.04
wt	362	36	
	MET		
mut	38	NR	0.04
wt	450	41	
	EPHA7		
mut	76	NR	0.04
wt	412	41	
	BCL6		
mut	18	NR	0.04
wt	470	41	
	SMO		
mut	32	NR	0.04
wt	456	46	
	CDK6		
mut	8	NR	0.04
wt	480	42	
	MED12		
mut	38	NR	0.04
wt	450	42	

N = number; OS = overall survival; NR = not reached.

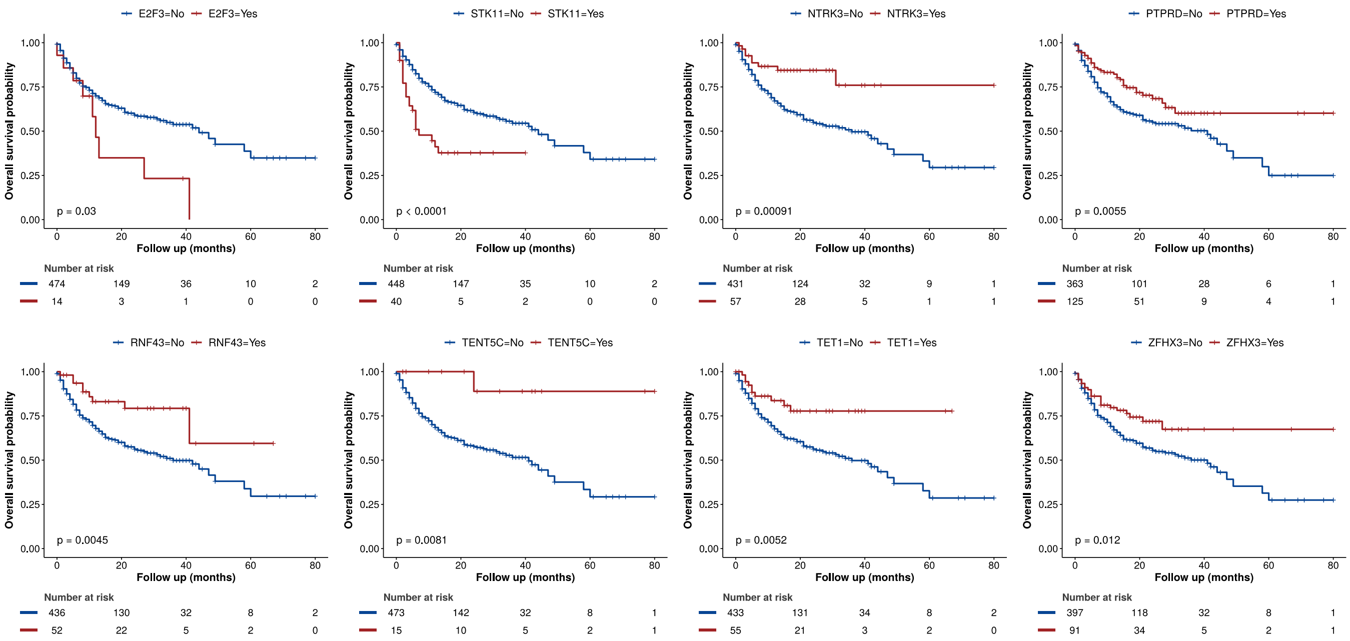

Figure S2. Kaplan–Meier (KM) curves for each gene related with survival after Cox multivariate analysis. Kaplan–Meier (KM) curves for overall survival for patients harboring gene mutations related with survival after Cox multivariate analysis (E2F3, STK11, NTRK3, PTPRD, RNF43, TENT5C, TET1, and ZFHX3) compared to the wild-type ones.

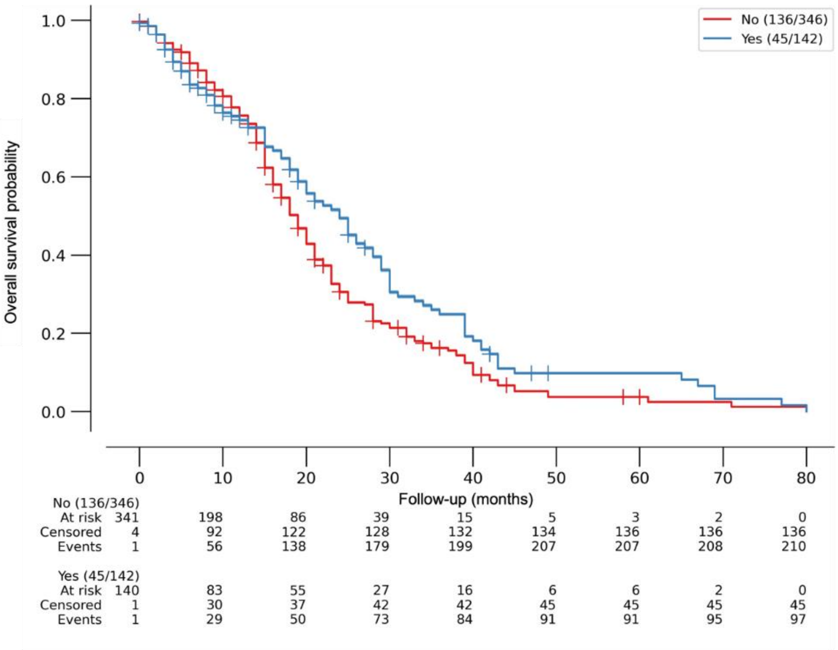

Figure S3. Effect of Microsatellite Instability (MSI) on overall survival after ICI treatment. Kaplan–Meier (KM) curves for TMB-high/MSI and with TMB-high/MSS tumors. Exploratory analyses performed in our TMB-high cohort identified that patients with TMB-high/MSI tumors exhibit better OS outcomes when compared with TMB-high/MSS tumors (median OS 42 vs 19 months; P < 0,05).

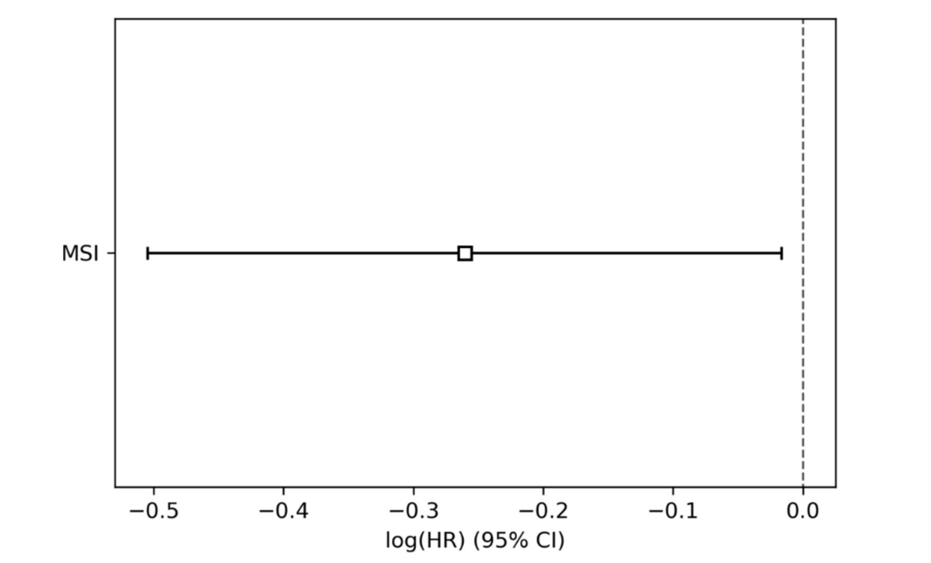

Figure S4. Effect of Microsatellite Instability (MSI) on overall survival after ICI treatment. HR for death was 0.77 (95% CI 0.60-0.98; P = 0.04).
